# Supplementary material for: Persistent multi-scale fluctuations shift European hydroclimate to its millennial boundaries
Source: Nat Commun. 2018 May 2;9:1767. doi: 10.1038/s41467-018-04207-7 (PMC5932079; doi:10.1038/s41467-018-04207-7)
Supplement: Supplementary file 1 — Supplementary Information [file 41467_2018_4207_MOESM1_ESM.pdf]

Persistent multi-scale fluctuations shift European hydroclimate to its millennial boundaries

Supplementary Material & Methods

Y. Markonis*[1], M. Hanel[1], P. Máca[1], J. Kyselý[1,2] and E. R. Cook[3]

[1]Faculty of Environmental Sciences, Czech University of Life Sciences Prague, Kamýcká 129, Praha – Suchdol, 165 00, Czech Republic

[2]Institute of Atmospheric Physics, Czech Academy of Sciences, Spořilov, Prague 141 31, Prague 141 31, Czech Republic

[3]Lamont-Doherty Earth Observatory, Palisades, New York 10964, USA

**Corresponding author: imarkonis@fzp.czu.cz*

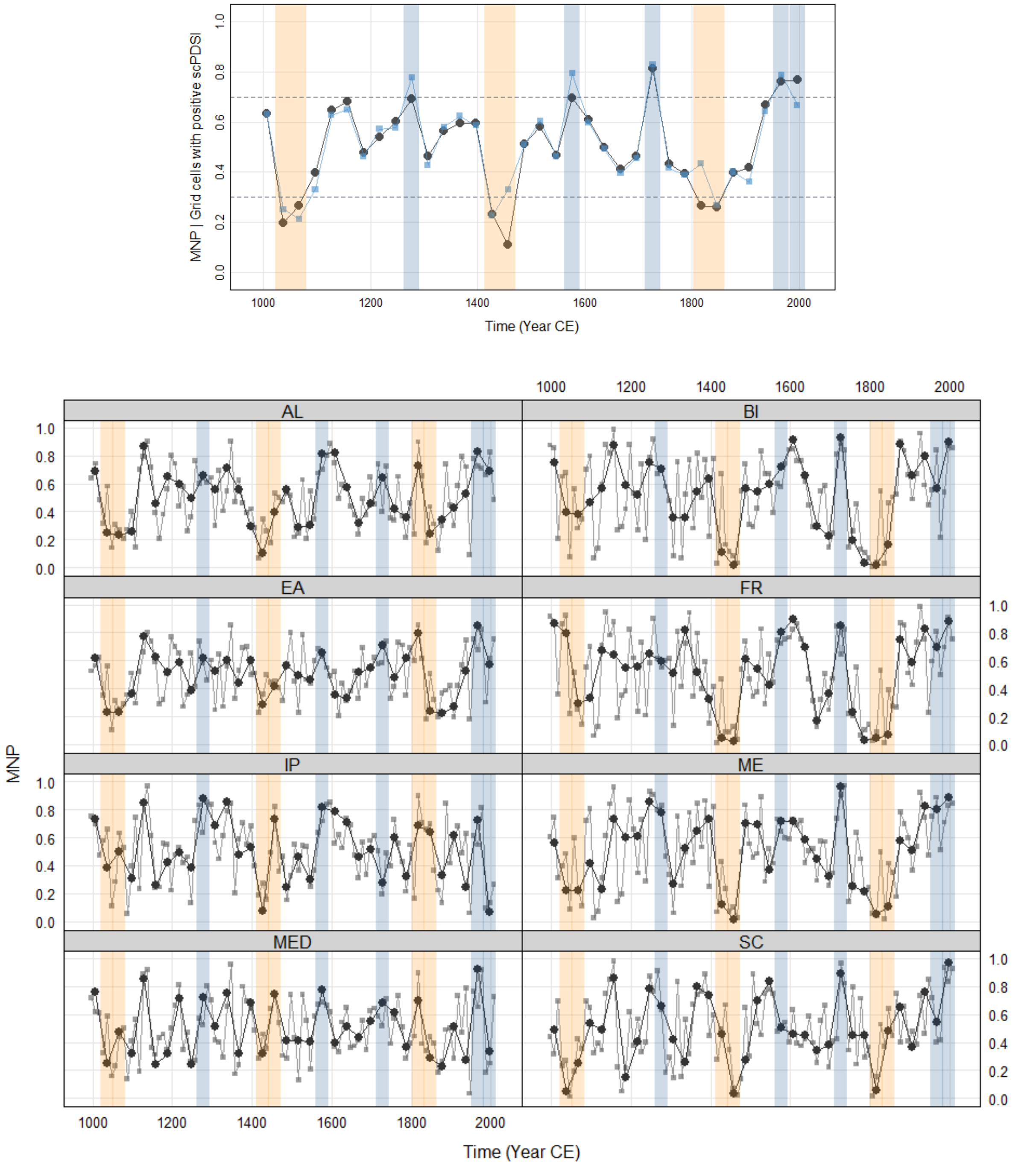

Figure 1. a. Mean of the non-exceedance probability (MNP) of the scPDSI for Europe at 30-year scale (black circled line) and percentage of grid cells that have positive scPDSI as a fraction of total number of grid cells (blue squared line). Grey dashed lines represent mean plus/minus one standard deviation of the scPDSI's MNP. **b.** MNP of the scPDSI for each PRUDENCE region (AL: Alps, BI: Britain Islands, EA: Eastern Europe, FR: France, IP: Iberian Peninsula, ME: Mid Europe, MED:Mediterranean, SC:Scandinavia) at 30-year scale (black) and 10-year scale (grey) and their relation to the dry (orange) and wet (blue) periods identified for the whole of Europe.

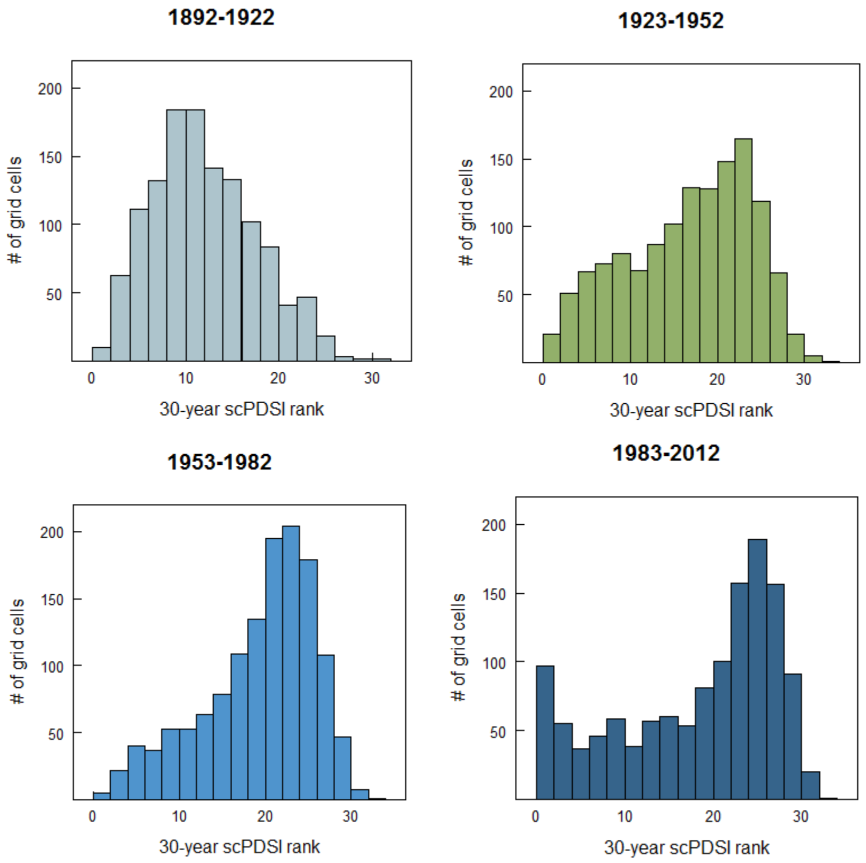

Figure 2. Empirical distribution of 30-year scPDSI presented in Figure 3, in terms of absolute ranks. A value of 1 corresponds to the driest 30-year interval observed, while a value of 34 to the wettest one.

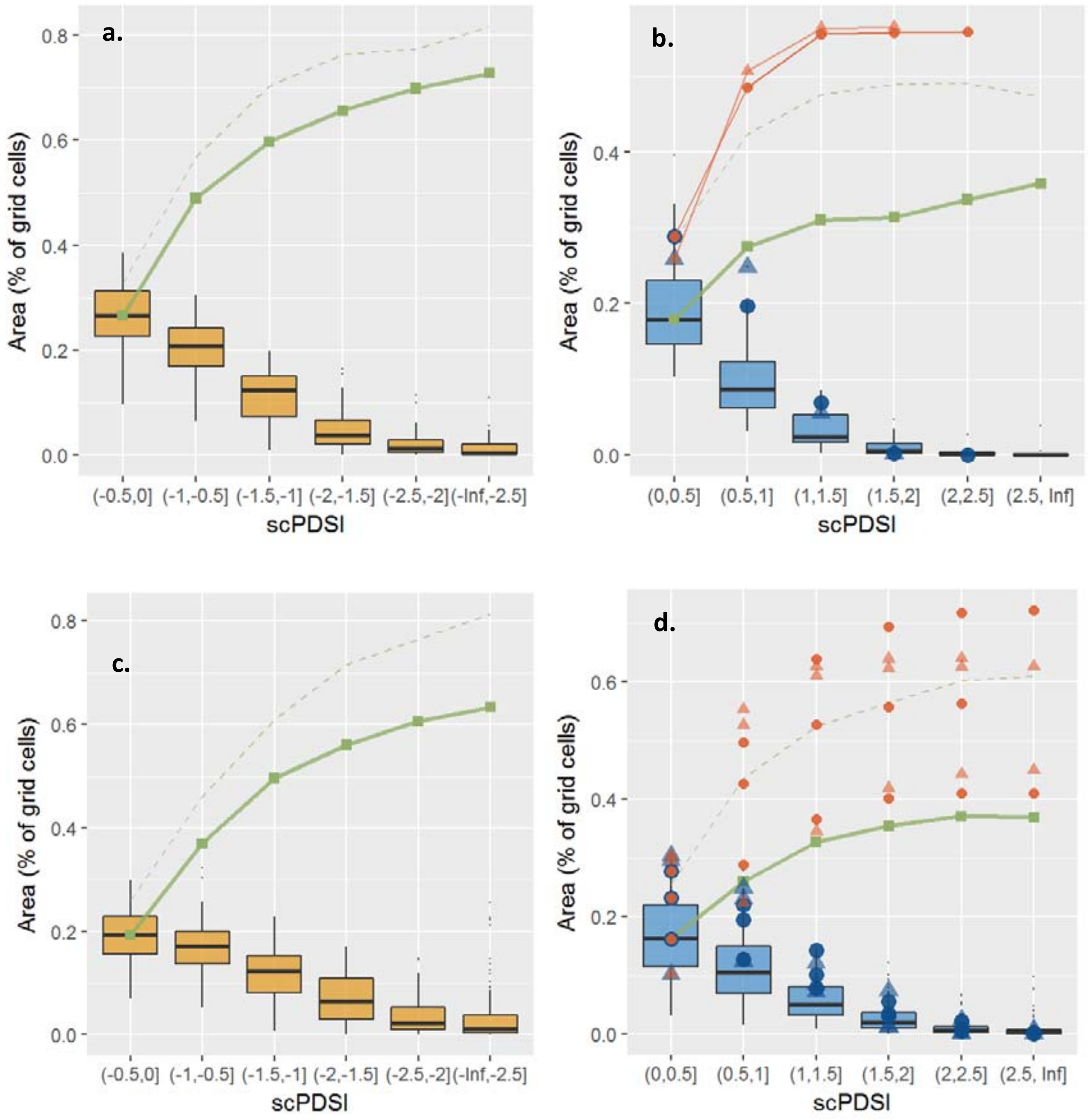

Figure 3. Investigation of areal extent, as percentage of total grid cell number, in conjunction to the drought severity, described by the original scPDSI values at each grid cell. The dry (*orange* boxplots) and wet (*blue* boxplots) conditions were examined for both 30-year (**a.**, **b.**) and 10-year scales (**c.**, **d.**). Cumulative area of dry/wet conditions has been also presented by adding the values of each successive bin (mean: *green square-pointed solid line*, 0.9 quantile: *green slashed line*). The comparison of the W4.EU interval (*blue points*: mean scPDSI, *orange points*: cumulative mean scPDSI; *triangles*: 1952-1982, *circles*: 1982-2012) with the other 10-/30-year periods, suggests that it was above the cumulative 0.9 quantile in the 30-year scale. This was due to its high values in the range between 0 and 1.5 and not as an outcome of extreme severe conditions (scPDSI > 2). Similar results appear for the 10-year scale, where all the decades of the last 60 years are found to be above the millennial mean, with three of them being in the top 0.1 quantile.

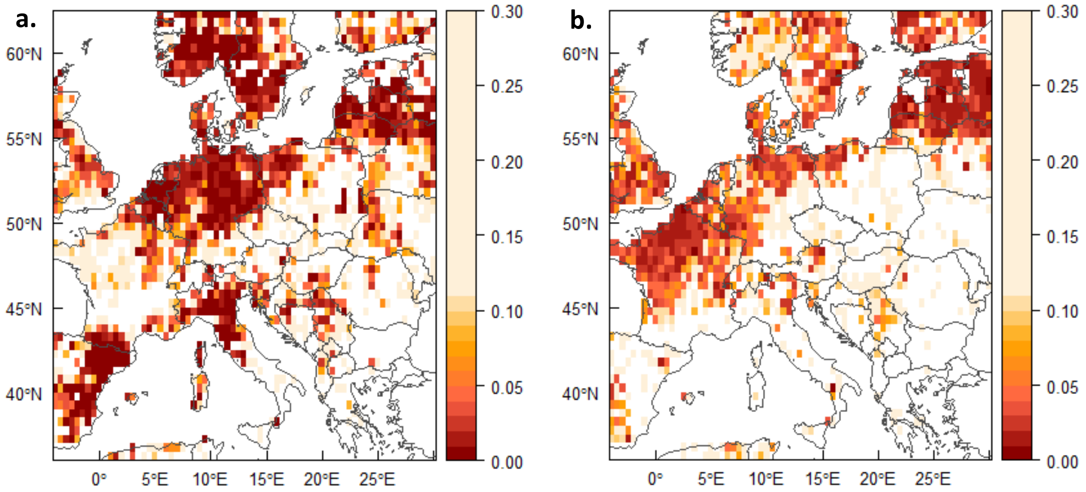

Figure 4. Statistical significance described as the rejection probability of the null hypothesis of the maximum likelihood estimator (Eq. 2), which is used in logistic regression as depicted in Figure 4 (**a.** Temperature and **b.** seasonal NAO).

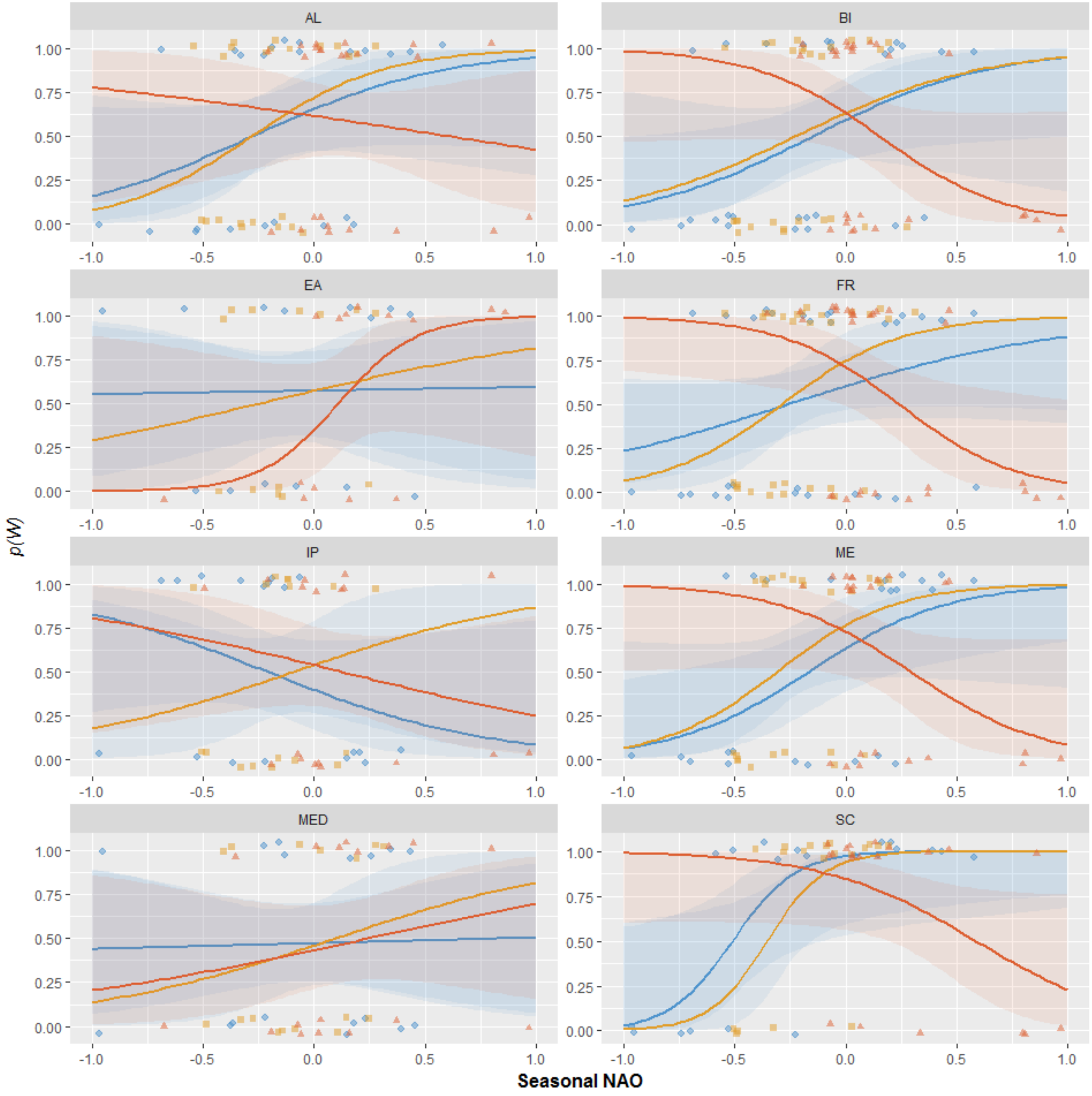

Figure 5. 10-year seasonal NAO values (*winter*: blue squares; *spring*: orange circles and *summer*: red triangles) versus empirical probability of wet conditions per each PRUDENCE region, i.e. extremely wet conditions are close to 1 while extremely dry close to 0. Continuous lines represent the corresponding logistic regression fit for each NAO variable. The random scattering of points over y axis was applied for clearer representation. Shaded area depicts the 0.95 confidence intervals.

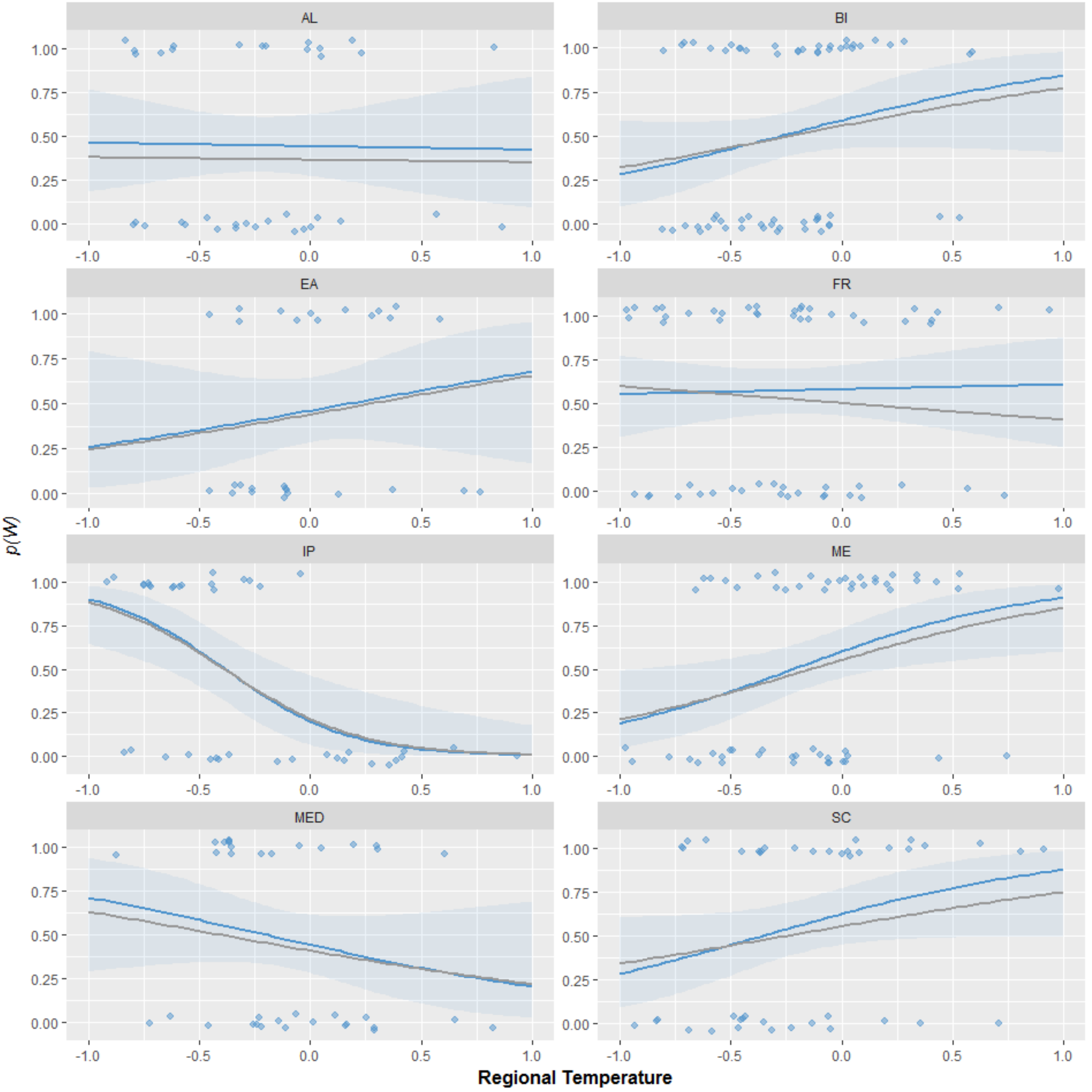

Figure 6. Similar to Figure S8, but for regional temperature (blue line). Removing the last 112 years (all records end at 1900 CE) does not significantly change the shapes of the logistic regression curves (grey line).

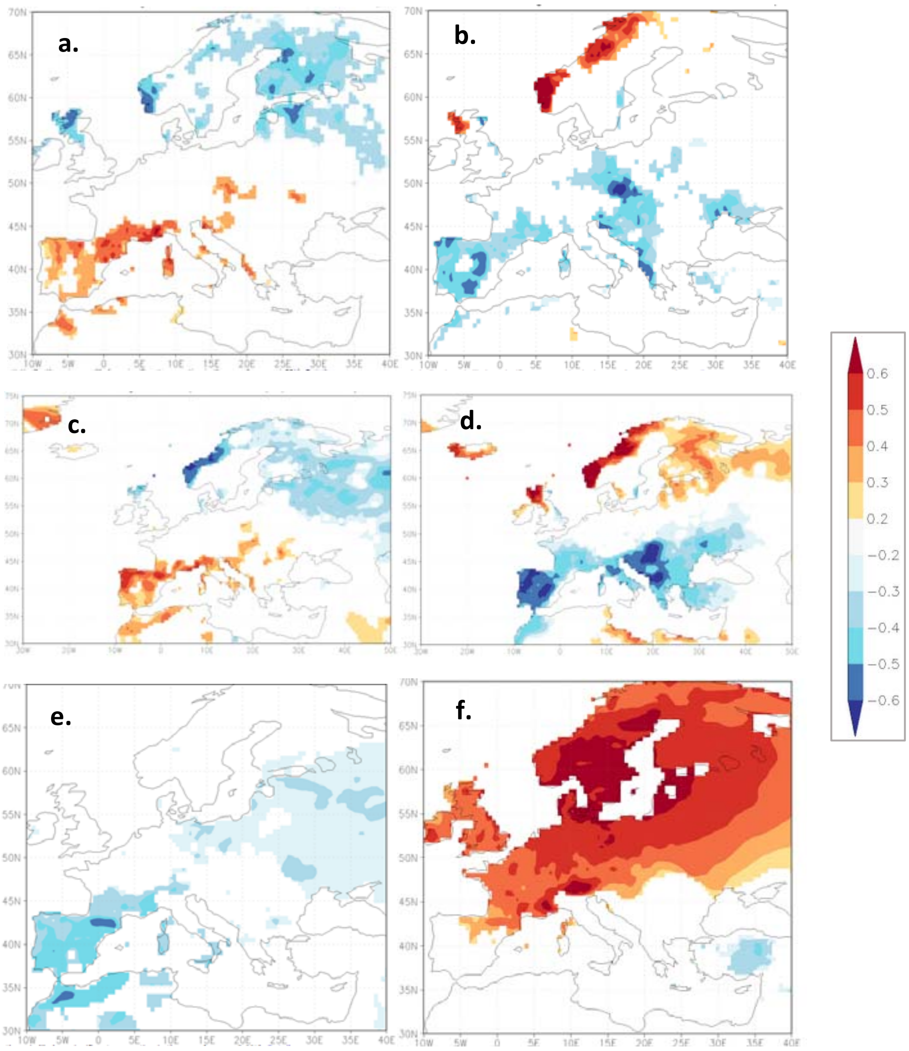

Figure 7. Correlation coefficients (0.9 significance) between CRU scPDSI 3.25 gridded dataset and annual **a.** SCA and **b.** AO. Similarly for precipitation (**c.**, **d.**) and temperature (**e.**, **f.**) (EOBS 15.0 dataset). Data extraction and analysis were performed in the KNMI explorer on-line platform.

Table 1. Absolute ranks of regional mean scPDSI. On the top row for each region is the 10-year rank (maximum 104), while in the succeeding is the 30-year rank (maximum 34). Top/lowest 20% percent of 30-year ranks is highlighted with blue/orange color.

	**1897**	**1907**	**1917**	**1927**	**1937**	**1947**	**1957**	**1967**	**1977**	**1987**	**1997**	**2007**
IP-10	36	67	80	51	78	1	56	50	93	3	6	21
IP-30	22			**6**			**29**			**1**		
FR-10	49	42	73	102	75	22	60	85	51	67	99	72
FR-30	16			**29**			25			**33**		
BI-10	67	40	64	101	51	43	50	87	22	60	95	96
BI-30	22			**29**			19			**32**		
ME-10	59	28	57	101	79	42	83	94	49	75	86	90
ME-30	15			**30**			**29**			**33**		
MED-10	52	31	91	48	94	1	92	70	102	6	14	88
MED-30	22			**4**			**34**			9		
AL-10	47	22	63	95	84	2	94	83	82	69	93	42
AL-30	13			18			**32**			25		
SC-10	42	58	34	85	43	90	79	48	38	99	92	100
SC-30	11			**28**			20			**34**		
EA-10	32	4	38	65	94	7	93	83	99	21	72	92
EA-30	**5**			16			**34**			25		

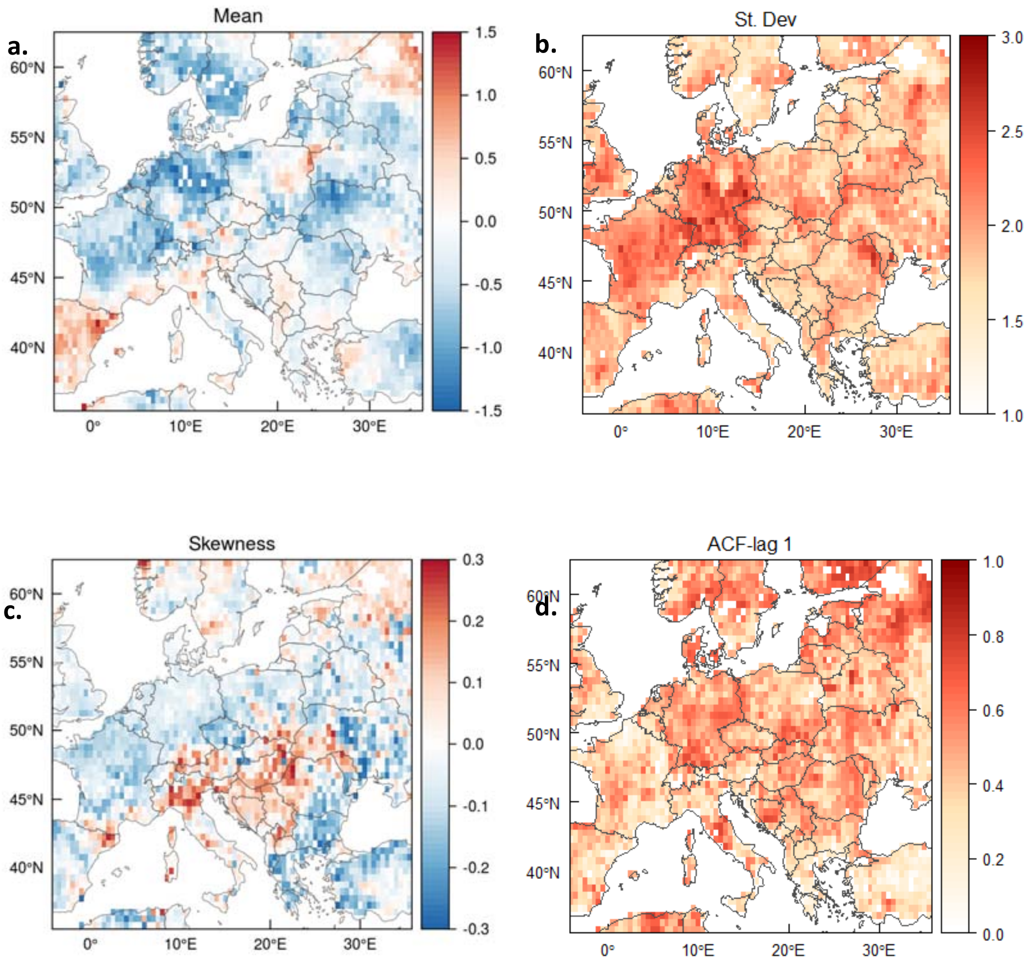

Figure 8. Spatial distribution of the statistical properties of the scPDSI: **a.** Mean, **b.** Standard deviation, **c.** Skewness, **d.** Autocorrelation coefficient (lag 1 year). It can be seen that the scPDSI mean deviates from zero; western mid and high latitudes demonstrate negative values, whereas the Iberian peninsula the sign inverses. These departures occur due to the calibration of OWDA to the instrumental period, i.e. 1928-1978, before it was applied to the tree-ring proxies. Thus, if the calibration period is wetter than the whole record, it would result to a negative mean value, whereas if it is dryer to a positive one. Therefore, to efficiently perform comparisons between different regions, and consequently different calibration conditions, the empirical quantiles of scPDSI were used.

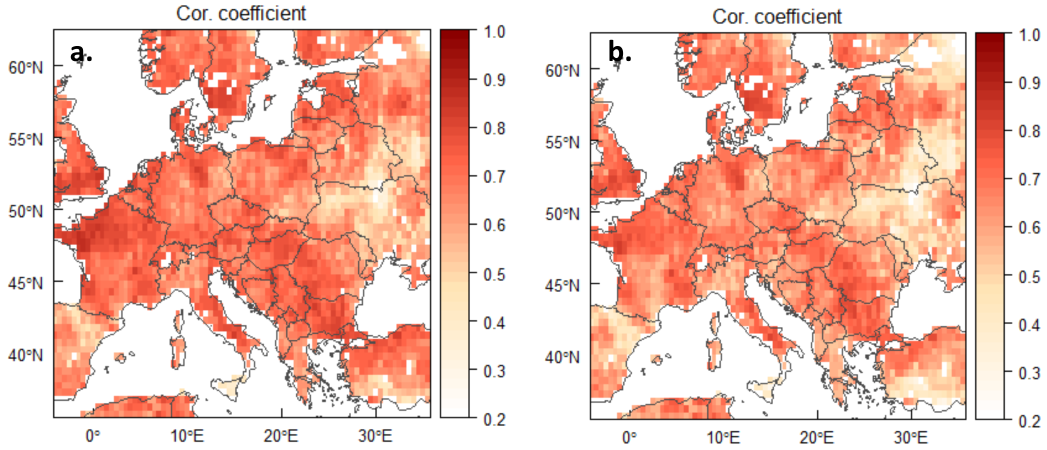

Figure 9. scPDSI correlation coefficient between OWDA (1900-2012) and CRU datasets for **a.** summer (ρ = 0.67) and **b.** annual time scale (ρ = 0.62). These values are likely to be biased upwards by some amount because the tree-ring estimates of the OWDA end in 1978. After appropriate scaling, the OWDA is extended to 2012 using instrumental scPDSI data.

Table 2. Recent paleoclimatic studies of regional drought. There is good agreement with other paleoclimatic studies as well. In the original paper, OWDA was found to be significantly correlated with the gridded precipitation reconstruction over Europe[1]. The same can be said for the Mediterranean[2], the Iberian Peninsula[3], Scandinavia[4,5], United Kingdom[6] and Czech lands[7]. Interestingly, the latter suggests intensive drying in that region during the last decades, which seems to contradict our results for the Central Europe region (Figure S3; ME). However, this is related to large spatial variability in Central Europe; if we further partition the ME region and focus on Czech lands (15-20ºE, 48-52ºN), then a dryness increase can be seen. Similar discrepancies were found for the Alps[8] and France[9], which were studies of specific sites.

Region	Citation	Index	Agreement	Discrepancy
**Mediterranean**	Cook et al. 2016	scPDSI	Analysis of OWDA in the Mediterranean	-
**Czech Lands**	Brázdil et al. 2016	SPEI, Z-index, scPDSI	Recent and 1800 dryness	1700-1750
**Scandinavia**	Seftigen et al. 2015	SPEI	Pluvial 1500-1600, Summer NAO	1600-1700 [subdecadal, both pluvial and dry]
**Scandinavia**	Drobyshev et al. 2015	LFY (large fire years)	Dry in 1800, Summer NAO	-
**Spain**	Tejedor et al. 2016	SPI	1750, 1803, 1808, 1870, 1980 droughts, 1760, 1820,1975 pluvials	1950 drought
**United Kingdom**	Spraggs et al. 2015	Runoff	1855, 1935 droughts	-
**Alpes (1 site)**	Kress et al. 2014	DRI	-	900-1100 drought, 1900-2000 pluvial
**France (2 sites)**	Labuhn et al. 2015	SPEI	-	Disagree

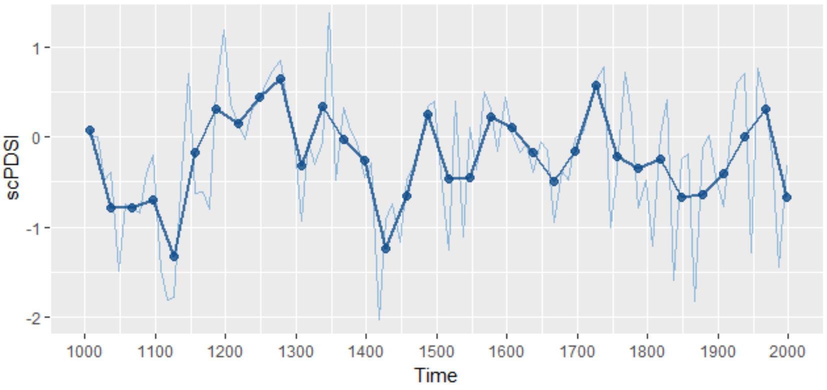

Figure 10. Hydroclimatic conditions (scPDSI) over the well-studied Czech region (15-20ºE, 48-52ºN), in 10-year (light blue line) and 30-year (dark blue line) scale.

References

1. Pauling, A., J. Luterbacher, C. Casty, and H. Wanner (2006), Five hundred years of gridded high-resolution precipitation reconstructions over Europe and the connection to large-scale circulation, Climate Dynamics, 26(4), 387–405.
2. Cook, B. I., K. J. Anchukaitis, R. Touchan, D. M. Meko, and E. R. Cook (2016), Spatiotemporal drought variability in the Mediterranean over the last 900 years, *Journal of Geophysical Research: Atmospheres*.
3. Tejedor, E., M. de Luis, J. M. Cuadrat, J. Esper, and M. Á. Saz (2016), Tree-ring-based drought reconstruction in the Iberian Range (east of Spain) since 1694, *International journal of biometeorology*, *60*(3), 361–372.
4. Drobyshev, I., Y. Bergeron, A. de Vernal, A. Moberg, A. A. Ali, and M. Niklasson (2016), Atlantic SSTs control regime shifts in forest fire activity of Northern Scandinavia, *Scientific reports*, *6*.
5. Seftigen, K., J. Björklund, E. R. Cook, and H. W. Linderholm (2015), A tree-ring field reconstruction of Fennoscandian summer hydroclimate variability for the last millennium, *Climate Dynamics*, *44*(11–12), 3141–3154.
6. Spraggs, G., L. Peaver, P. Jones, and P. Ede (2015), Re-construction of historic drought in the Anglian Region (UK) over the period 1798–2010 and the implications for water resources and drought management, *Journal of Hydrology*, *526*, 231–252.
7. Brázdil, R., P. Dobrovolný, M. Trnka, U. Büntgen, L. Řezníčková, O. Kotyza, and others (2016), Documentary and instrumental-based drought indices for the Czech Lands back to AD 1501, *Climate Research*, *70*(2–3), 103–117.
8. Kress, A., S. Hangartner, H. Bugmann, U. Büntgen, D. C. Frank, M. Leuenberger, R. T. Siegwolf, and M. Saurer (2014), Swiss tree rings reveal warm and wet summers during medieval times, *Geophysical Research Letters*, *41*(5), 1732–1737.
9. Labuhn, I., V. Daux, O. Girardclos, M. Stievenard, M. Pierre, and V. Masson-Delmotte (2016), French summer droughts since 1326 CE: a reconstruction based on tree ring cellulose δ 18 O, *Climate of the Past*, *12*(5), 1101–1117.
